# Supplementary material for: Hot spot prediction in protein-protein interactions by an ensemble system
Source: BMC Syst Biol. 2018 Dec 31;12(Suppl 9):132. doi: 10.1186/s12918-018-0665-8 (PMC6311905; doi:10.1186/s12918-018-0665-8)
Supplement: Supplementary file 2 — All relevant MATLAB code. A simple Matlab implement of our predictor. (ZIP 19,742 kb) [file 12918_2018_665_MOESM2_ESM.zip › Additional file 2/Read ASA/ASA website/dbMPIKT/3MBWA.html]

 NetSurfP - Protein Surface Accessibility and Secondary Structure Predictions

  

|  |  |  |
| --- | --- | --- |
|  |  | NetSurfP - Protein Surface Accessibility and Secondary Structure Predictions  Technical University of Denmark |

  


---

```
# For publication of results, please cite:
# A generic method for assignment of reliability scores applied to solvent accessibility predictions.
# Bent Petersen, Thomas Nordahl Petersen, Pernille Andersen, Morten Nielsen and Claus Lundegaard
# BMC Structural Biology 2009, 9:51 doi:10.1186/1472-6807-9-51
#
# Column 1: Class assignment - B for buried or E for Exposed - Threshold: 25% exposure, but not based on RSA
# Column 2: Amino acid
# Column 3: Sequence name
# Column 4: Amino acid number
# Column 5: Relative Surface Accessibility - RSA
# Column 6: Absolute Surface Accessibility
# Column 7: Z-fit score for RSA prediction
# Column 8: Probability for Alpha-Helix
# Column 9: Probability for Beta-strand
# Column 10: Probability for Coil
E A  3MBWA                  1    0.834  91.852  -0.266   0.003   0.003   0.994
E P  3MBWA                  2    0.526  74.696  -1.791   0.018   0.019   0.964
E E  3MBWA                  3    0.707 123.460  -0.762   0.053   0.043   0.903
E H  3MBWA                  4    0.497  90.313  -1.053   0.053   0.043   0.903
E H  3MBWA                  5    0.459  83.528  -0.946   0.053   0.043   0.903
E H  3MBWA                  6    0.399  72.651  -0.898   0.052   0.084   0.864
E H  3MBWA                  7    0.370  67.358  -0.587   0.052   0.084   0.864
E H  3MBWA                  8    0.373  67.831  -0.892   0.113   0.087   0.800
E H  3MBWA                  9    0.361  65.648  -0.465   0.113   0.087   0.800
E D  3MBWA                 10    0.356  51.285  -0.913   0.113   0.087   0.800
B Y  3MBWA                 11    0.186  39.812   0.113   0.052   0.084   0.864
E D  3MBWA                 12    0.504  72.612   0.473   0.052   0.084   0.864
B I  3MBWA                 13    0.059  10.989   0.589   0.113   0.043   0.844
E P  3MBWA                 14    0.315  44.656   0.410   0.115   0.016   0.868
E T  3MBWA                 15    0.290  40.265  -0.272   0.268   0.043   0.689
B T  3MBWA                 16    0.046   6.422   0.070   0.453   0.248   0.299
B E  3MBWA                 17    0.090  15.688  -0.888   0.578   0.229   0.194
B N  3MBWA                 18    0.075  10.995   0.001   0.680   0.265   0.055
B L  3MBWA                 19    0.033   5.987   0.023   0.746   0.197   0.057
B Y  3MBWA                 20    0.068  14.574   0.417   0.725   0.163   0.112
B F  3MBWA                 21    0.059  11.861  -0.527   0.807   0.137   0.056
B Q  3MBWA                 22    0.055   9.859   0.098   0.779   0.100   0.120
B G  3MBWA                 23    0.083   6.564  -0.144   0.701   0.107   0.192
B A  3MBWA                 24    0.064   7.075  -0.952   0.561   0.047   0.393
B M  3MBWA                 25    0.095  19.009  -0.561   0.455   0.046   0.498
E D  3MBWA                 26    0.252  36.328   0.403   0.455   0.046   0.498
B A  3MBWA                 27    0.176  19.351  -1.291   0.455   0.046   0.498
B A  3MBWA                 28    0.129  14.205  -0.630   0.354   0.048   0.598
E Q  3MBWA                 29    0.288  51.383   0.334   0.278   0.093   0.628
B G  3MBWA                 30    0.164  12.875  -1.118   0.199   0.152   0.649
E K  3MBWA                 31    0.266  54.716   0.526   0.216   0.235   0.548
B E  3MBWA                 32    0.171  29.891  -0.083   0.231   0.330   0.439
B V  3MBWA                 33    0.122  18.705   0.715   0.252   0.423   0.325
B V  3MBWA                 34    0.161  24.684   0.518   0.268   0.505   0.227
B L  3MBWA                 35    0.057  10.382   0.282   0.159   0.516   0.325
B L  3MBWA                 36    0.099  18.054   0.016   0.148   0.418   0.435
B D  3MBWA                 37    0.193  27.768  -1.031   0.066   0.296   0.638
B F  3MBWA                 38    0.161  32.252  -0.381   0.056   0.142   0.802
E A  3MBWA                 39    0.505  55.651  -0.443   0.113   0.087   0.800
E A  3MBWA                 40    0.442  48.730  -0.721   0.118   0.150   0.732
B A  3MBWA                 41    0.261  28.740  -0.626   0.199   0.152   0.649
E G  3MBWA                 42    0.405  31.905  -1.172   0.199   0.152   0.649
B G  3MBWA                 43    0.253  19.903  -1.112   0.135   0.317   0.548
E E  3MBWA                 44    0.415  72.431  -0.188   0.069   0.386   0.545
B L  3MBWA                 45    0.092  16.809   0.094   0.021   0.451   0.528
B G  3MBWA                 46    0.262  20.604  -0.443   0.022   0.552   0.426
B W  3MBWA                 47    0.095  22.727  -0.197   0.023   0.655   0.322
B L  3MBWA                 48    0.208  38.066   0.255   0.021   0.756   0.223
B T  3MBWA                 49    0.212  29.377  -1.259   0.023   0.655   0.322
B H  3MBWA                 50    0.219  39.782   0.954   0.005   0.262   0.733
E P  3MBWA                 51    0.281  39.874   0.218   0.004   0.085   0.910
E Y  3MBWA                 52    0.468  99.947  -0.650   0.005   0.045   0.951
E G  3MBWA                 53    0.709  55.798  -1.670   0.018   0.019   0.964
E K  3MBWA                 54    0.599 123.255  -0.640   0.018   0.019   0.964
E G  3MBWA                 55    0.417  32.810  -0.467   0.018   0.019   0.964
B W  3MBWA                 56    0.106  25.397  -0.078   0.056   0.142   0.802
E D  3MBWA                 57    0.396  57.006  -0.351   0.074   0.484   0.442
E L  3MBWA                 58    0.328  60.002  -0.532   0.074   0.484   0.442
B M  3MBWA                 59    0.136  27.214   0.177   0.074   0.484   0.442
B Q  3MBWA                 60    0.221  39.488  -1.025   0.074   0.484   0.442
B N  3MBWA                 61    0.311  45.545  -1.968   0.021   0.451   0.528
E I  3MBWA                 62    0.268  49.617  -0.106   0.021   0.451   0.528
E M  3MBWA                 63    0.344  68.874  -1.128   0.021   0.279   0.699
E N  3MBWA                 64    0.562  82.233  -1.788   0.018   0.088   0.893
E D  3MBWA                 65    0.633  91.244  -2.832   0.018   0.047   0.935
E M  3MBWA                 66    0.316  63.132  -0.690   0.018   0.088   0.893
B P  3MBWA                 67    0.218  30.892  -0.788   0.004   0.138   0.858
B I  3MBWA                 68    0.122  22.570  -0.062   0.004   0.514   0.481
B Y  3MBWA                 69    0.157  33.636  -0.308   0.003   0.718   0.279
B M  3MBWA                 70    0.111  22.291  -0.240   0.002   0.816   0.182
B Y  3MBWA                 71    0.085  18.165   0.006   0.002   0.816   0.182
B S  3MBWA                 72    0.099  11.626  -1.824   0.001   0.900   0.099
B V  3MBWA                 73    0.150  23.116  -0.636   0.018   0.846   0.136
B C  3MBWA                 74    0.034   4.774  -0.025   0.023   0.655   0.322
E N  3MBWA                 75    0.302  44.154   0.393   0.021   0.451   0.528
B V  3MBWA                 76    0.219  33.599  -1.114   0.020   0.205   0.775
E M  3MBWA                 77    0.503 100.730  -1.651   0.018   0.088   0.893
E S  3MBWA                 78    0.522  61.225  -1.276   0.005   0.045   0.951
E G  3MBWA                 79    0.602  47.385  -2.826   0.005   0.015   0.979
E D  3MBWA                 80    0.537  77.425  -1.528   0.018   0.019   0.964
E Q  3MBWA                 81    0.337  60.260  -0.659   0.018   0.019   0.964
E D  3MBWA                 82    0.336  48.374  -0.874   0.018   0.047   0.935
B N  3MBWA                 83    0.080  11.741  -0.755   0.018   0.088   0.893
B W  3MBWA                 84    0.069  16.570  -0.177   0.021   0.451   0.528
B L  3MBWA                 85    0.048   8.825   0.021   0.023   0.655   0.322
B R  3MBWA                 86    0.167  38.289  -0.122   0.022   0.552   0.426
B T  3MBWA                 87    0.079  10.916  -0.166   0.021   0.279   0.699
E N  3MBWA                 88    0.395  57.799   0.178   0.020   0.205   0.775
B W  3MBWA                 89    0.205  49.182  -0.106   0.021   0.451   0.528
B V  3MBWA                 90    0.080  12.281   0.146   0.023   0.655   0.322
E Y  3MBWA                 91    0.438  93.643  -0.066   0.021   0.451   0.528
E R  3MBWA                 92    0.396  90.798  -0.781   0.019   0.141   0.840
E G  3MBWA                 93    0.508  39.980  -0.090   0.018   0.047   0.935
E E  3MBWA                 94    0.558  97.552  -0.623   0.018   0.047   0.935
B A  3MBWA                 95    0.082   9.036  -1.241   0.004   0.197   0.799
E E  3MBWA                 96    0.289  50.541   0.623   0.004   0.514   0.481
B R  3MBWA                 97    0.195  44.586   1.249   0.001   0.900   0.099
B I  3MBWA                 98    0.032   5.994   0.380   0.001   0.959   0.040
B F  3MBWA                 99    0.132  26.452   0.970   0.001   0.959   0.040
B I  3MBWA                100    0.035   6.401   0.261   0.001   0.959   0.040
B E  3MBWA                101    0.146  25.436   0.855   0.001   0.959   0.040
B L  3MBWA                102    0.035   6.372   0.376   0.001   0.959   0.040
E K  3MBWA                103    0.326  67.140   1.855   0.001   0.959   0.040
B F  3MBWA                104    0.042   8.429   0.567   0.001   0.900   0.099
B T  3MBWA                105    0.144  19.917   0.527   0.001   0.900   0.099
B V  3MBWA                106    0.056   8.592   0.333   0.018   0.846   0.136
B R  3MBWA                107    0.269  61.601   0.455   0.022   0.552   0.426
E D  3MBWA                108    0.278  40.031  -0.021   0.066   0.296   0.638
B C  3MBWA                109    0.036   5.026  -0.946   0.052   0.084   0.864
E N  3MBWA                110    0.486  71.136  -0.578   0.053   0.043   0.903
E S  3MBWA                111    0.393  46.036  -1.531   0.018   0.047   0.935
B F  3MBWA                112    0.236  47.305  -0.099   0.018   0.019   0.964
E P  3MBWA                113    0.552  78.300  -1.352   0.018   0.019   0.964
E G  3MBWA                114    0.545  42.892  -2.387   0.018   0.019   0.964
E G  3MBWA                115    0.493  38.807  -2.178   0.018   0.019   0.964
E A  3MBWA                116    0.466  51.309  -1.889   0.018   0.047   0.935
E S  3MBWA                117    0.313  36.695  -0.721   0.018   0.047   0.935
B S  3MBWA                118    0.277  32.441   0.033   0.118   0.150   0.732
B C  3MBWA                119    0.043   6.079   0.340   0.199   0.152   0.649
E K  3MBWA                120    0.336  69.033   1.109   0.307   0.165   0.527
E E  3MBWA                121    0.331  57.843  -0.674   0.321   0.252   0.427
B T  3MBWA                122    0.253  35.105   0.069   0.321   0.252   0.427
B F  3MBWA                123    0.061  12.343   0.242   0.231   0.330   0.439
B N  3MBWA                124    0.235  34.477   0.021   0.231   0.330   0.439
B L  3MBWA                125    0.056  10.217   0.939   0.252   0.423   0.325
B Y  3MBWA                126    0.106  22.567   0.524   0.169   0.612   0.219
B Y  3MBWA                127    0.094  19.981   0.781   0.169   0.612   0.219
B A  3MBWA                128    0.153  16.872   0.875   0.159   0.516   0.325
B E  3MBWA                129    0.199  34.695   0.305   0.148   0.418   0.435
B S  3MBWA                130    0.107  12.482   0.243   0.125   0.227   0.648
E D  3MBWA                131    0.282  40.593   0.137   0.113   0.087   0.800
E L  3MBWA                132    0.409  74.815   0.142   0.113   0.043   0.844
E D  3MBWA                133    0.478  68.880  -0.338   0.113   0.043   0.844
E Y  3MBWA                134    0.462  98.623  -0.077   0.113   0.043   0.844
B G  3MBWA                135    0.277  21.808  -1.195   0.053   0.043   0.903
E T  3MBWA                136    0.442  61.361  -0.512   0.053   0.043   0.903
E N  3MBWA                137    0.580  84.868  -0.569   0.053   0.043   0.903
B F  3MBWA                138    0.170  34.199  -0.614   0.053   0.043   0.903
E Q  3MBWA                139    0.482  86.157  -0.540   0.018   0.047   0.935
E K  3MBWA                140    0.510 104.928  -0.345   0.053   0.043   0.903
E R  3MBWA                141    0.504 115.347  -0.937   0.053   0.043   0.903
E L  3MBWA                142    0.400  73.258   0.057   0.053   0.043   0.903
B F  3MBWA                143    0.165  33.095  -0.808   0.125   0.227   0.648
B T  3MBWA                144    0.216  29.945   0.409   0.074   0.484   0.442
B K  3MBWA                145    0.260  53.400   0.479   0.079   0.592   0.329
B I  3MBWA                146    0.051   9.453   0.806   0.087   0.683   0.230
B D  3MBWA                147    0.125  18.056  -0.367   0.087   0.683   0.230
B T  3MBWA                148    0.190  26.395   0.047   0.079   0.592   0.329
B I  3MBWA                149    0.048   8.843   0.636   0.074   0.484   0.442
B A  3MBWA                150    0.157  17.345   0.721   0.064   0.216   0.721
B P  3MBWA                151    0.198  28.054  -0.863   0.018   0.088   0.893
E D  3MBWA                152    0.412  59.326  -0.485   0.018   0.047   0.935
E E  3MBWA                153    0.361  63.102  -0.404   0.019   0.141   0.840
B I  3MBWA                154    0.193  35.724  -1.414   0.021   0.279   0.699
B T  3MBWA                155    0.151  20.902   0.223   0.021   0.451   0.528
E V  3MBWA                156    0.348  53.441  -0.791   0.022   0.359   0.619
E S  3MBWA                157    0.375  43.950  -0.983   0.019   0.141   0.840
E S  3MBWA                158    0.406  47.618  -2.046   0.018   0.088   0.893
E D  3MBWA                159    0.565  81.445  -1.660   0.018   0.047   0.935
E F  3MBWA                160    0.305  61.294  -1.582   0.018   0.019   0.964
E E  3MBWA                161    0.607 106.025  -1.058   0.018   0.019   0.964
E A  3MBWA                162    0.433  47.750  -1.479   0.018   0.088   0.893
E R  3MBWA                163    0.344  78.890  -0.182   0.021   0.451   0.528
E H  3MBWA                164    0.462  84.074  -0.216   0.002   0.816   0.182
B V  3MBWA                165    0.183  28.189   0.878   0.011   0.918   0.071
E K  3MBWA                166    0.386  79.338   0.381   0.001   0.900   0.099
B L  3MBWA                167    0.175  32.061   0.621   0.002   0.816   0.182
E N  3MBWA                168    0.344  50.318  -0.291   0.004   0.514   0.481
B V  3MBWA                169    0.224  34.490   0.285   0.003   0.718   0.279
E E  3MBWA                170    0.319  55.799  -0.029   0.001   0.900   0.099
E E  3MBWA                171    0.289  50.471  -0.181   0.001   0.900   0.099
B R  3MBWA                172    0.257  58.922   0.701   0.018   0.846   0.136
E S  3MBWA                173    0.494  57.955  -0.960   0.003   0.718   0.279
B V  3MBWA                174    0.243  37.349  -0.490   0.004   0.420   0.576
B G  3MBWA                175    0.298  23.445  -0.703   0.005   0.262   0.733
E P  3MBWA                176    0.501  71.092  -0.745   0.021   0.279   0.699
B L  3MBWA                177    0.192  35.228  -0.451   0.020   0.205   0.775
E T  3MBWA                178    0.347  48.115  -0.606   0.018   0.088   0.893
E R  3MBWA                179    0.464 106.187  -0.001   0.018   0.047   0.935
E K  3MBWA                180    0.419  86.250   0.561   0.018   0.088   0.893
B G  3MBWA                181    0.068   5.367   0.042   0.021   0.451   0.528
B F  3MBWA                182    0.030   5.921   0.414   0.018   0.846   0.136
B Y  3MBWA                183    0.060  12.758   0.458   0.011   0.918   0.071
B L  3MBWA                184    0.030   5.585   0.540   0.011   0.918   0.071
B A  3MBWA                185    0.020   2.226   0.986   0.018   0.846   0.136
B F  3MBWA                186    0.033   6.563  -0.103   0.021   0.756   0.223
B Q  3MBWA                187    0.169  30.255   0.846   0.021   0.451   0.528
B D  3MBWA                188    0.248  35.780  -0.443   0.064   0.216   0.721
E I  3MBWA                189    0.260  48.119   0.396   0.052   0.084   0.864
B G  3MBWA                190    0.147  11.545  -1.709   0.052   0.084   0.864
B A  3MBWA                191    0.036   3.945  -0.217   0.125   0.227   0.648
B C  3MBWA                192    0.025   3.496   0.604   0.159   0.516   0.325
B V  3MBWA                193    0.064   9.775   0.885   0.273   0.587   0.140
B A  3MBWA                194    0.035   3.824  -0.812   0.273   0.587   0.140
B L  3MBWA                195    0.040   7.251  -0.009   0.391   0.466   0.143
B L  3MBWA                196    0.075  13.787  -0.470   0.391   0.466   0.143
B S  3MBWA                197    0.149  17.510  -0.898   0.273   0.587   0.140
B V  3MBWA                198    0.046   7.070   0.206   0.273   0.587   0.140
B R  3MBWA                199    0.169  38.678   0.859   0.387   0.545   0.068
B V  3MBWA                200    0.068  10.498  -0.121   0.273   0.587   0.140
B Y  3MBWA                201    0.111  23.699   0.222   0.273   0.587   0.140
B Y  3MBWA                202    0.095  20.366   0.095   0.273   0.587   0.140
E K  3MBWA                203    0.319  65.659   0.411   0.159   0.516   0.325
E K  3MBWA                204    0.364  74.875   0.656   0.231   0.330   0.439
B C  3MBWA                205    0.064   9.042   0.568   0.184   0.043   0.773
B P  3MBWA                206    0.264  37.433  -0.308   0.622   0.015   0.363
E E  3MBWA                207    0.461  80.607  -0.034   0.717   0.014   0.269
B L  3MBWA                208    0.148  27.026  -0.210   0.717   0.014   0.269
B L  3MBWA                209    0.085  15.545  -0.401   0.717   0.014   0.269
E Q  3MBWA                210    0.357  63.707  -0.356   0.717   0.014   0.269
B G  3MBWA                211    0.153  12.057  -1.236   0.622   0.015   0.363
B L  3MBWA                212    0.056  10.199   0.404   0.561   0.047   0.393
B A  3MBWA                213    0.114  12.574  -1.543   0.502   0.102   0.396
E H  3MBWA                214    0.313  56.971   0.208   0.386   0.097   0.517
B F  3MBWA                215    0.102  20.491   0.707   0.191   0.086   0.723
E P  3MBWA                216    0.296  42.017  -0.331   0.113   0.043   0.844
E E  3MBWA                217    0.450  78.545  -0.655   0.053   0.043   0.903
B T  3MBWA                218    0.204  28.295  -0.920   0.113   0.087   0.800
B I  3MBWA                219    0.189  35.039  -0.125   0.113   0.087   0.800
E A  3MBWA                220    0.353  38.879  -0.575   0.053   0.043   0.903
E G  3MBWA                221    0.396  31.134  -1.538   0.053   0.043   0.903
E S  3MBWA                222    0.501  58.682  -1.780   0.058   0.017   0.925
E D  3MBWA                223    0.564  81.244  -1.424   0.053   0.043   0.903
B A  3MBWA                224    0.258  28.476  -0.875   0.056   0.142   0.802
E P  3MBWA                225    0.361  51.297  -0.286   0.021   0.279   0.699
B S  3MBWA                226    0.177  20.780  -0.357   0.023   0.655   0.322
B L  3MBWA                227    0.168  30.669   0.470   0.018   0.846   0.136
B A  3MBWA                228    0.151  16.596   0.248   0.011   0.918   0.071
E T  3MBWA                229    0.381  52.789   0.103   0.001   0.900   0.099
B V  3MBWA                230    0.107  16.384   0.137   0.003   0.718   0.279
E A  3MBWA                231    0.487  53.623  -0.413   0.005   0.336   0.660
B G  3MBWA                232    0.261  20.564  -1.708   0.005   0.336   0.660
E T  3MBWA                233    0.405  56.118   0.185   0.003   0.718   0.279
B C  3MBWA                234    0.073  10.207   0.195   0.004   0.616   0.381
B V  3MBWA                235    0.226  34.736   1.004   0.022   0.359   0.619
E D  3MBWA                236    0.636  91.676  -0.512   0.019   0.141   0.840
E H  3MBWA                237    0.468  85.147  -1.304   0.018   0.047   0.935
B A  3MBWA                238    0.119  13.147  -3.546   0.018   0.088   0.893
E V  3MBWA                239    0.528  81.107  -0.961   0.021   0.451   0.528
E V  3MBWA                240    0.545  83.751  -0.445   0.021   0.451   0.528
E P  3MBWA                241    0.534  75.775  -0.820   0.020   0.205   0.775
E P  3MBWA                242    0.523  74.242  -1.141   0.018   0.088   0.893
E G  3MBWA                243    0.671  52.847  -2.406   0.018   0.047   0.935
E G  3MBWA                244    0.637  50.156  -1.693   0.018   0.047   0.935
E E  3MBWA                245    0.522  91.141  -1.853   0.018   0.047   0.935
E E  3MBWA                246    0.445  77.794  -0.438   0.004   0.085   0.910
B P  3MBWA                247    0.107  15.141  -2.135   0.005   0.336   0.660
B R  3MBWA                248    0.236  54.136   0.013   0.003   0.718   0.279
B M  3MBWA                249    0.096  19.130  -0.987   0.002   0.816   0.182
B H  3MBWA                250    0.257  46.730   0.192   0.018   0.846   0.136
B C  3MBWA                251    0.033   4.591  -0.875   0.004   0.514   0.481
E A  3MBWA                252    0.333  36.741  -0.675   0.019   0.141   0.840
E V  3MBWA                253    0.572  87.901  -2.455   0.018   0.047   0.935
E D  3MBWA                254    0.623  89.803  -1.247   0.018   0.047   0.935
B G  3MBWA                255    0.177  13.938  -2.168   0.019   0.141   0.840
E E  3MBWA                256    0.486  84.887  -0.485   0.022   0.359   0.619
B W  3MBWA                257    0.196  47.138  -1.147   0.021   0.451   0.528
B L  3MBWA                258    0.218  39.952  -0.987   0.021   0.451   0.528
B V  3MBWA                259    0.178  27.312   0.096   0.022   0.359   0.619
B P  3MBWA                260    0.280  39.704  -0.672   0.022   0.359   0.619
E I  3MBWA                261    0.432  79.864  -1.500   0.019   0.141   0.840
E G  3MBWA                262    0.462  36.375  -2.012   0.018   0.088   0.893
E Q  3MBWA                263    0.406  72.494   0.687   0.021   0.279   0.699
B C  3MBWA                264    0.040   5.574  -0.779   0.022   0.552   0.426
E L  3MBWA                265    0.292  53.520   0.620   0.023   0.655   0.322
B C  3MBWA                266    0.037   5.251   0.043   0.022   0.552   0.426
E Q  3MBWA                267    0.426  76.173   0.929   0.021   0.279   0.699
E A  3MBWA                268    0.509  56.092  -0.781   0.018   0.088   0.893
E G  3MBWA                269    0.397  31.275  -1.885   0.018   0.047   0.935
E Y  3MBWA                270    0.313  66.995  -0.546   0.021   0.279   0.699
E E  3MBWA                271    0.382  66.700   0.020   0.022   0.359   0.619
E K  3MBWA                272    0.586 120.622  -0.449   0.020   0.205   0.775
E V  3MBWA                273    0.570  87.640   0.067   0.018   0.047   0.935
E E  3MBWA                274    0.695 121.347  -0.688   0.018   0.019   0.964
E D  3MBWA                275    0.537  77.353   0.489   0.018   0.047   0.935
E A  3MBWA                276    0.416  45.876   1.925   0.021   0.451   0.528
B C  3MBWA                277    0.025   3.538  -0.859   0.021   0.756   0.223
E Q  3MBWA                278    0.435  77.673   0.939   0.021   0.756   0.223
E A  3MBWA                279    0.514  56.654  -0.031   0.022   0.552   0.426
E C  3MBWA                280    0.272  38.203   0.421   0.019   0.141   0.840
E S  3MBWA                281    0.490  57.475  -1.800   0.004   0.085   0.910
E P  3MBWA                282    0.428  60.790  -0.682   0.004   0.085   0.910
B G  3MBWA                283    0.228  17.951  -2.130   0.005   0.336   0.660
E F  3MBWA                284    0.262  52.603   0.003   0.004   0.616   0.381
B F  3MBWA                285    0.101  20.311  -0.101   0.002   0.816   0.182
E K  3MBWA                286    0.262  53.955  -0.068   0.021   0.756   0.223
E F  3MBWA                287    0.342  68.559  -0.820   0.023   0.655   0.322
E E  3MBWA                288    0.337  58.787  -0.895   0.022   0.359   0.619
E A  3MBWA                289    0.466  51.331  -1.762   0.004   0.138   0.858
E S  3MBWA                290    0.523  61.296  -1.937   0.018   0.047   0.935
E E  3MBWA                291    0.579 101.169  -1.719   0.018   0.047   0.935
E S  3MBWA                292    0.543  63.581  -0.914   0.018   0.088   0.893
E P  3MBWA                293    0.514  72.866   0.583   0.020   0.205   0.775
B C  3MBWA                294    0.072  10.067  -2.164   0.021   0.451   0.528
E L  3MBWA                295    0.345  63.133   0.358   0.022   0.552   0.426
E E  3MBWA                296    0.572  99.859   0.675   0.021   0.451   0.528
B C  3MBWA                297    0.043   6.009   0.307   0.004   0.085   0.910
E P  3MBWA                298    0.356  50.545  -0.463   0.005   0.045   0.951
E E  3MBWA                299    0.567  99.090  -1.202   0.018   0.019   0.964
E H  3MBWA                300    0.498  90.623  -1.362   0.018   0.047   0.935
E T  3MBWA                301    0.356  49.336  -2.251   0.018   0.088   0.893
E L  3MBWA                302    0.440  80.509  -0.506   0.019   0.141   0.840
B P  3MBWA                303    0.214  30.409  -1.124   0.018   0.047   0.935
E S  3MBWA                304    0.448  52.470  -0.314   0.018   0.047   0.935
E P  3MBWA                305    0.519  73.589  -0.509   0.018   0.047   0.935
E E  3MBWA                306    0.629 109.974  -0.377   0.018   0.047   0.935
E G  3MBWA                307    0.383  30.174  -0.948   0.018   0.047   0.935
B A  3MBWA                308    0.143  15.726  -1.564   0.019   0.141   0.840
E T  3MBWA                309    0.427  59.267  -0.380   0.022   0.359   0.619
E S  3MBWA                310    0.516  60.452   0.730   0.004   0.514   0.481
B C  3MBWA                311    0.059   8.340   0.572   0.004   0.616   0.381
E E  3MBWA                312    0.425  74.282   1.630   0.018   0.846   0.136
B C  3MBWA                313    0.085  11.976  -0.244   0.021   0.756   0.223
E E  3MBWA                314    0.452  78.964   0.646   0.022   0.359   0.619
E E  3MBWA                315    0.730 127.618   0.780   0.018   0.088   0.893
E G  3MBWA                316    0.410  32.243  -1.020   0.052   0.084   0.864
B F  3MBWA                317    0.161  32.313   0.882   0.021   0.451   0.528
B F  3MBWA                318    0.185  37.069  -0.165   0.023   0.655   0.322
B R  3MBWA                319    0.262  60.044   0.276   0.023   0.655   0.322
E A  3MBWA                320    0.233  25.710   0.183   0.021   0.279   0.699
E P  3MBWA                321    0.616  87.339   0.263   0.018   0.088   0.893
E Q  3MBWA                322    0.715 127.717  -0.850   0.018   0.019   0.964
E D  3MBWA                323    0.552  79.572  -1.332   0.005   0.015   0.979
E P  3MBWA                324    0.499  70.865  -0.879   0.003   0.003   0.994
E A  3MBWA                325    0.535  58.990  -0.059   0.005   0.015   0.979
E S  3MBWA                326    0.458  53.713  -1.143   0.005   0.045   0.951
E M  3MBWA                327    0.429  85.843  -0.346   0.004   0.085   0.910
E P  3MBWA                328    0.496  70.425  -0.692   0.005   0.045   0.951
B C  3MBWA                329    0.258  36.279  -1.317   0.018   0.047   0.935
E T  3MBWA                330    0.725 100.585  -1.256   0.003   0.003   0.994

Explain the output.  Go back.
```

---
